# A New Electrochemical Sensor Based on Task-Specific Ionic Liquids-Modified Palm Shell Activated Carbon for the Determination of Mercury in Water Samples

**DOI:** 10.3390/s140713102

**Published:** 2014-07-21

**Authors:** Ahmed Abu Ismaiel, Mohamed Kheireddine Aroua, Rozita Yusoff

**Affiliations:** Chemical Engineering Department, University of Malaya, Kuala Lumpur 50603, Malaysia; E-Mails: abu_ismaiel@hotmail.com (A.A.I.); ryusoff@um.edu.my (R.Y.)

**Keywords:** palm shell activated carbon, ionic liquid, TOMATS, ion selective electrode, mercury

## Abstract

In this study, a potentiometric sensor composed of palm shell activated carbon modified with trioctylmethylammonium thiosalicylate (TOMATS) was used for the potentiometric determination of mercury ions in water samples. The proposed potentiometric sensor has good operating characteristics towards Hg (II), including a relatively high selectivity; a Nernstian response to Hg (II) ions in a concentration range of 1.0 × 10^−9^ to 1.0 × 10^−2^ M, with a detection limit of 1 × 10^−10^ M and a slope of 44.08 ± 1.0 mV/decade; and a fast response time (∼5 s). No significant changes in electrode potential were observed when the pH was varied over the range of 3–9. Additionally, the proposed electrode was characterized by good selectivity towards Hg (II) and no significant interferences from other cationic or anionic species.

## 1. Introduction

Mercury is a major water pollutant of high concern, producing severe ailments in living beings, including mental retardation. It is a toxic bio-accumulative environmental pollutant that affects the nervous system and it is released into the environment through industrial, agricultural and other anthropogenic processes. Interests in the determination of trace amount of mercury ions have significantly increased during the past few years due to growing environmental concerns. Several analytical techniques, including, cold vapor atomic absorption spectrometry (CV-AAS) [1–3], inductively coupled plasma optical emission spectrometry (ICP-OES) [4,5], X-ray fluorescence spectrometry [6], inductively coupled plasma mass spectrometry (ICP-MS) [7,8] and cold vapour atomic fluorescence spectrometry (CV-AFS) [9–11] have been applied for the determination of trace amounts of mercury in analytical samples. These methods have good sensitivity, and well controlled experimental conditions. However, they have several disadvantages, such as the use of expensive apparatus, complicated operation, high operation and maintenance costs, and the requirement of well controlled experimental conditions. Because of their advantages in terms of low cost, easy fabrication, simplicity, sensitivity and fast response time, potentiometric sensors based on ion selective electrodes have attracted much attention in electro-analytical chemistry and have been successfully used to determine trace levels of hazardous pollutants such as mercury [12–29].

Ion selective electrodes (ISEs) are potentiometric sensors used to measure some of the most hazardous analytes in environmental laboratory and point-of-care analysers. Despite their easy fabrication, simple usage, and low cost, ISEs suffer from low response sensitivity, interference by a number of metal ions and short lifetimes. As a result, the development of new ISE materials that can address some of these limitations is a worthwhile and challenging topic of research. The ultimate goals of this study are to increase the sensitivity and selectivity of the proposed electrode by minimizing the previously mentioned undesirable electrode processes. Additionally, the application of plasticizer-free electrodes can eliminate the leaching of the electrode solvent and sensing components, thus improving the electrode lifetime.

Carbon is a very important electrode material and is widely used due to its low cost, easy functionalisation, great versatility, broad potential window, and chemical inertness. Various forms of carbons, such as glassy carbon, impregnated graphite, carbon fibres, carbon films, carbon nanotubes, and activated carbon, could be used as electrode materials. Palm shell, a waste product of palm kernel oil production, represents an important group of carbonaceous materials with unique mechanical, physical, and electrochemical properties [30].

Room temperature ionic liquids are salts having very low melting temperature close to room temperature. Room temperature ionic liquids have become an extremely popular theme in recent electrochemical sensing research, due to their large electrochemical window, high conductivity, non-volatility, low toxicity and good electrochemical stability. Recently, new ion selective sensors based on room temperature ionic liquids have been developed [19,31–33].

**Figure 1.**
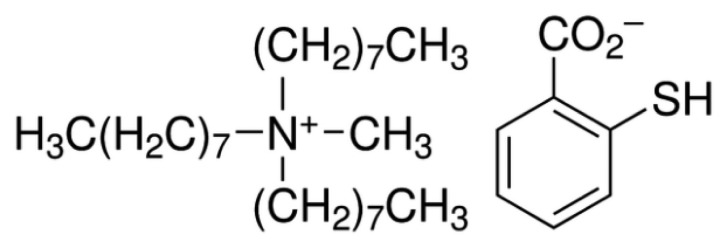
Chemical structure of TOMATS.

In this work palm shell activated carbon was modified with trioctylmethylammonium thiosalicylate (TOMATS) to act as a new potentiometric sensor for determination of Hg (II) ion in water samples. It is worth mentioning that TOMATS acts as ionophore as well as plasticizer. To the best of our knowledge this is the first study reporting the use of TOMATS (structure as shown in Figure 1) for the determination of mercury ions in water samples. TOMATS was previously shown to be a very good ligand for Hg (II) [30] which makes it a potential ionophore in a potentiometric sensor.

## 2. Experimental Section

### 2.1. Chemicals and Reagents

Analytical reagent grade chemicals and distilled, de-ionized water were used to prepare all aqueous solutions. Commercially available granular palm shell activated carbon (PSAC) was provided by Bravo Green Sdn. Bhd, Malaysia. Activated carbon powder with particle sizes <40 μm were used throughout the potentiometric experiments. PSAC was washed with distilled water to remove fines and dirt; and was dried in an oven at 110 °C for 24 h. The pH of the solutions was adjusted by adding appropriate amounts of concentrated hydrochloric acid (1 M HCl) and/or sodium hydroxide (2 M NaOH). Metal salts were purchased from Merck (Selangor, Malaysia), and aqueous metal solutions were prepared by dissolving appropriate quantities of metal salts in de-ionized water. trioctylmethylammonium thiosalicylate (TOMATS) was purchased from Sigma-Aldrich (Kuala Lumpur, Malaysia).

### 2.2. Apparatus

All potentiometric measurements were made using a pH/ion meter (Metrohm-781, Filderstadt, Germany) and pH Module (Metrohm-867), permitting real-time potential data collection using the proposed electrode in conjunction with a double junction Ag/AgCl reference electrode. The temperature of the cell holder was maintained at 25 °C and measured under constant stirring with a magnetic stirring bar at 180 rpm.

The electrochemical cell used in this study was constructed as follows:

Ag(s), AgCl(s) | KCl(3 M sat.)| |Sample solution| modified palm shell activated carbon paste electrode.

Metal ion sample concentration was analyzed by inductivity coupled plasma optical emission spectrometer ICP-OES. (model ICP optima 7000DV, PerkinElmer, Waltham, MA, USA).

### 2.3. Modified Palm Shell Activated Carbon Paste Electrode Preparations and Potential Measurements

Modified palm shell activated carbon paste was prepared by hand mixing the determined quantities of palm shell activated carbon powder and TOMATS. The optimal paste quality was obtained by mixing 0.15 g PSAC and 0.15 g TOMATS in the ratio 1:1 (w/w). The constituents were thoroughly hand mixed in a 50 mm Petri dish to produce the optimal paste quality and then the paste was poured and packed into empty glassy carbon electrode (5 mm diameter) connected to the pH/ion meter by a thin copper wire to produce an electrical contact. The composite surface was polished on weighing paper until the surface displayed a shiny appearance. The surface was rinsed carefully with double-distilled water prior to each experiment. The electrode is stored in a desiccator when it is not in use to avoid adsorption of contaminants.

The potentiometric measurements were conducted as follows: the modified carbon paste electrode and reference electrode were placed in 50 mL of a stirred, 0.1 M Hg (II) solution until the potential reading was constant. The standard addition method was used to investigate the electrode response characteristics. Mercury salt standard solutions were added so that the mercury concentration ranged between 10^−10^ and 10^−1^ M. A suitable volume (0.2–100 μL) of mercury standards was pipetted into 50 mL of water in a measuring beaker and the potential measured in the appropriate way for the ion to be measured (*i.e.*, with stirring and sufficient time for stable reading). The potential readings were recorded after each addition when stable values had been obtained. The concentration of solutions was checked by ICP.

The electrode potential of the electrochemical cell *E*_cell_ is described by the following Nernst equation:

(1) $$E_{\text{cell}}=E_{\text{cons}}+2.303\frac{RT}{zF}\log a$$

where *E*_cons_ is a constant term (the sum of the standard potential and liquid junction potential), *R* is the ideal gas constant, *T* is the absolute temperature, *F* is the Faraday constant, *z* is the charge of the ion, and *a* is the activity of the ion. At low concentrations, the activity value *a* can be replaced with the concentration value *C*. The prelogarithmic factor $2.303\frac{RT}{zF}$ is obtained from the slope (*S*) of the plot of *E*_cell_ *versus* log C, and the equation becomes:

(2) $$E_{\text{cell}}=E_{\text{cons}}+S\log\text{C}$$

Potentiometric selectivity of this electrode towards different cations was calculated with the matched potential method (MPM) [34]. In this method, the activity of Hg (II) was increased from a_i_ = 1.0 × 10^−5^ M (primary ion) to á_i_ = 5.0 × 10^−5^ M, and the corresponding potential change (ΔE) was measured. Then a solution of an interfering ion (a_j_) in the concentration range of 1.0 × 10^−1^–1.0 × 10^−2^ M was added to a new primary ion (á_i_) until the same potential change (ΔE) was recorded. The selectivity factor, k_ij_^pot^, was calculated for each interferent using the following equation:

(3) $$k_{ij}^{pot}=(a_{i}\prime -a_{i})/a_{j}$$

## 3. Results and Discussion

### 3.1. Response of the Electrode

**Figure 2.**
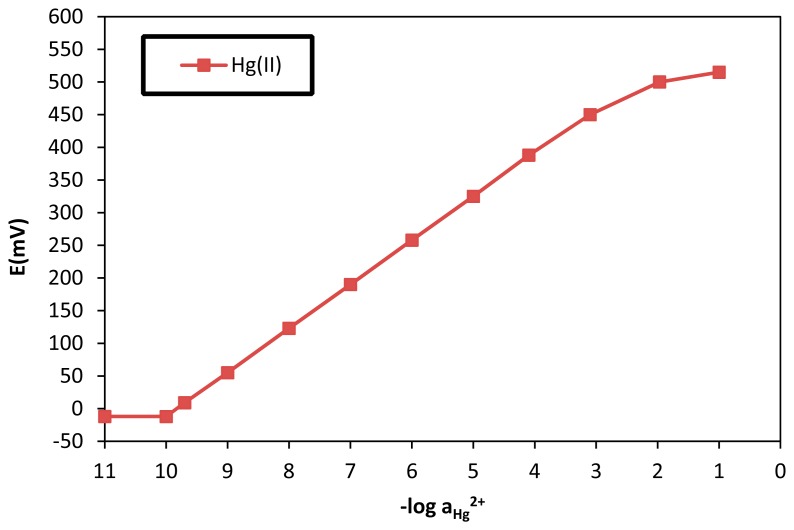
Calibration curve for a modified palm shell activated carbon paste electrode over a wide range of Hg (II) activities.

The calibration for the developed electrode over a wide range of solution Hg (II) activities is shown in Figure 2. The slope of the calibration curve (44.08 mV/dec) is close to that predicted theoretically by the Nernst equation (58.16 mV/dec for monovalent cations), which may be attributed to the formation of monovalent mercury complexes on the electrode surface. This finding indicates that the electrode was sensitive to Hg (II) over a wide range of Hg (II) activities (1 × 10^−9^–1 × 10^−2^ M).

In addition, the electrode showed a linear response over this range of activities, with a departure from linearity (*i.e.*, loss of sensitivity) at activities lower than 10^−9^ M Hg (II). The unique sensitivity and selectivity towards Hg (II) obtained for this electrode is due to the coordinate interaction between TOMATS and Hg (II) ions, which may be explained by the chelating effect of the ortho-positioned carboxylate group on the TOMATS molecule impregnated on palm shell activated carbon in addition to the known formation of metal-thiolates [35].

TOMATS which was used as the solvent mediator and plasticizer, have certain desirable properties and characteristics, such as high lipophilicity, high molecular weight, and low vapour pressure. Additionally, their viscosities and dielectric constants were adequate for the construction of an ion selective electrode with desirable analytical properties, such as selectivity, sensitivity, fast response, and long lifetime. The critical response characteristics of the proposed electrode were evaluated according to IUPAC recommendations [36].

### 3.2. Effect of pH on Electrode Response

The pH of each solution was verified, and its effect on the electrode potential at various metal concentrations was studied. For this purpose, several Hg (II) concentrations (1.0 × 10^−6^ M, 1.0 × 10^−4^ M and 1.0 × 10^−3^ M) were prepared, and the potential variations of the electrode over a pH range of 1–12 were followed. The pH was adjusted by adding small volumes of hydrochloric acid (1 M) and/or sodium hydroxide (2 M) to the sample solution.

**Figure 3.**
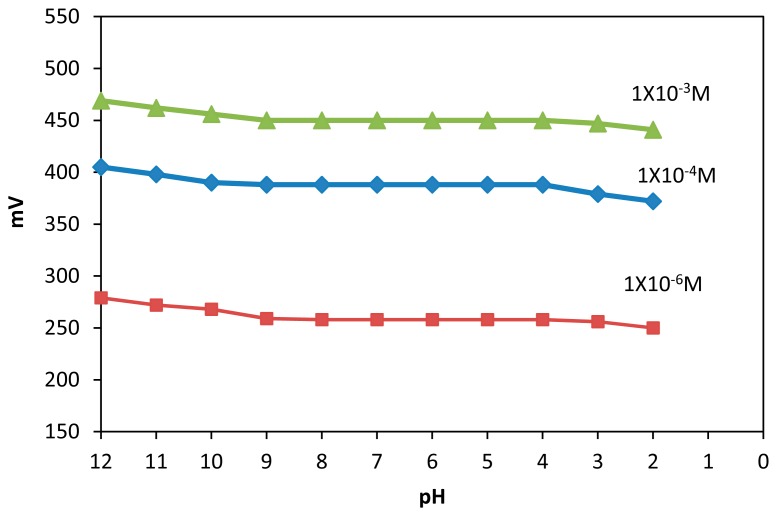
Effect of pH on the potential response of Hg (II) palm shell activated carbon paste electrode.

The results, shown in Figure 3, indicate that the potential remained constant in the pH range of 3–9, which can be used as the working pH range of the proposed electrode. However, outside this range, the electrode responses changed slightly. The diminished potential at pH > 9 was due to the interference of OH^−^ on the surface. The response at pH < 3 seemed ascribable to the competitive binding of protons to the ligands on the electrode surface.

### 3.3. Potentiometric Selectivity Coefficients

It is well known that the selectivity behavior of an electrode is one of the most important factors in its evaluation, which is measured in terms of the selectivity coefficient. The selectivity coefficient not only depends on ion charge and concentration, but it can also be affected by the type of interaction between the ion and the ionophore. The selectivity factor, log *k*^pot^ is a measure of the preference of ion selective electrode for interfering ion relative to the primary ion to be measured. A selectivity factor log *k*^pot^ below 1 indicates that the preference is for the primary ion.

The values of the selectivity coefficients, listed in Table 1, reflect a very high selectivity of this electrode for mercury (II) ion over most of the tested species *j*. Ag^+^, Pb^2+^ and Cu^2+^ caused only slight interference. However, they do not cause any interference at low concentration. As shown in Table 1, it can be observed, that the proposed electrode based on TOMATS exhibited better selectivity for mercury (II) ion over a wide variety of other metal ions.

### 3.4. Dynamic Response Time

**Figure 4.**
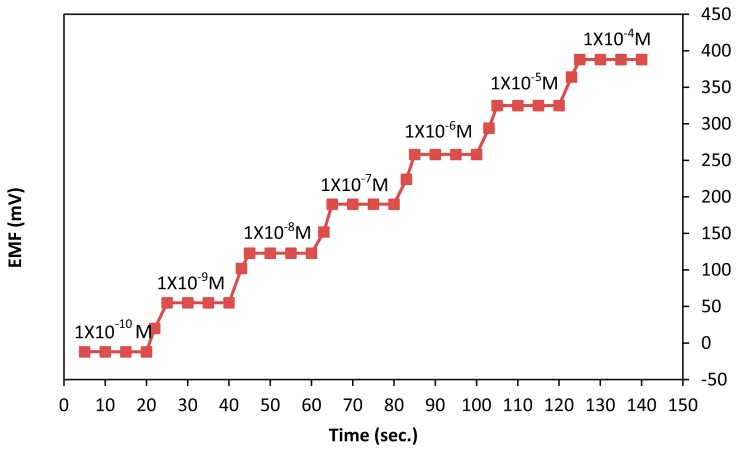
Response time of the electrode obtained by successive increase of Hg (II) ion.

The response time of the electrode is one of the most important characteristics of the ion selective electrode. According to IUPAC recommendations, the response time of an ion selective electrode is defined as the time between the addition of the analyte to the sample solution and the time when limiting potential has reached its steady state value within ±1 mV. In this study, the response time of the electrode was tested by measuring the time required to achieve a steady state potential (within ±1 mV of the final equilibrium value) after successive immersion in a series of Hg (II) ions. The results, shown in Figure 4 indicate that the response time of the electrode was approximately 5 s for the solution of mercury ion in the concentration range of 1 × 10^−8^–1 × 10^−4^ M. This result is probably due to the fast complexation of Hg (II) ions by the TOMATS molecule dispersed in the palm shell activated carbon paste matrix.

### 3.5. Electrode Life Time

The life time of the electrode depends on the distribution coefficient of the electrode compositions between the aqueous phase and the electrode phase. Accordingly, the life time of the electrode must depend on the electrode components.

In this work, the life time of the electrode was determined by performing periodic calibrations with standard solutions and calculating the slopes over Hg (II) ion concentration ranges of 1 × 10^−9^ to 1 × 10^−2^ M. The obtained results showed that the lifetime of the present electrode was over 90 days (Table 2). During this time, the detection limit of the electrode remained almost constant and the slope of the electrode response decreases from 44.08 to 42.17 mV per decade. Therefore, the electrode can be used for at least 3 months, without a considerable change in their response characteristic towards Hg (II) ions.

### 3.6. Comparison of the Response for the Proposed Hg (II) Electrode with other Reported Electrodes

The comparison of the performance of the proposed electrode with that of some recently developed electrodes for Hg (II) determination is given in Table 3.

As clearly shown in this table the sensor proposed in this work has excellent performance in terms of response time, linear range, Nernstian slope, and detection limit. The proposed electrodes showed improved performance characteristics relative to conventional electrodes. This improvement presumably originates from the electrode composition.

### 3.7. Analytical Applications

The proposed electrode was applied for determination of Hg (II) in real drinking water sample. The results of Hg (II) content measured by proposed electrode were compared with those obtained by ICP. Table 4 shows that Hg (II) concentration values obtained by proposed electrode were similar to those obtained by ICP, with deviations below 2% for all samples.

## 4. Conclusions

The results presented herein demonstrate the utility of TOMATS as both plasticizer and ionophore in the preparation of new ion selective electrodes for the determination of mercury ions in water samples. The proposed electrode had fast response for detection of mercury ions. The proposed electrode has good operating characteristics towards Hg (II), including a relatively high selectivity; a Nernstian response to Hg (II) ions in a concentration range of 1.0 × 10^−9^ to 1.0 × 10^−2^ M, with a detection limit of 1 × 10^−10^ M and a slope of 44.08 ± 1.0 mV/decade; and a fast response time (∼5 s). No significant changes in electrode potential were observed when the pH was varied over the range of 3–9. The electrode was successfully applied for the determination of mercury content in drinking water samples. These characteristics and the typical applications presented in this work make the electrode suitable for measuring the mercury content in real samples without a significant interaction from other cationic or anionic species.

## Figures and Tables

**Table 1.** Selectivity coefficient values of various interfering ions with Hg (II) selective electrode using matched potential method (MPM).

Interferent ion, *j*	−log *k*^pot^_Hg2+,_*_j_*	Interferent ion, *j*	−log *k*^pot^_Hg2+,_ *_j_*
Cu^2+^	3.05	Na^+^	4.89
Cd^2+^	3.64	K^+^	4.64
Ca^2+^	4.89	Ni^2+^	3.72
Mg^2+^	4.48	Cr^3+^	4.10
Zn^2+^	3.96	Co^2+^	3.33
Al^3+^	3.92	Ag^+^	3.05
Fe^3+^	4.24	Pb^2+^	3.02

**Table 2.** Mercury electrode response during 90 days.

Time Period (day)	Slope (mV/decade)	Limit of Detection (M)
1	44.08	1 × 10^−10^
7	44.08	1 × 10^−10^
20	44.08	1 × 10^−10^
30	43.86	1 × 10^−10^
50	43.29	1 × 10^−10^
70	42.86	1 × 10^−9^
90	42.17	1 × 10^−9^

**Table 3.** Comparison of the proposed Hg electrode with previously reported electrodes.

Ionophore	Slope (mV/decade)	Linear range (mol·L^−1^)	Detection limit (mol·L^−1^)	Response time (s)	Reference
TOMATS	44.08 ± 1.0	1 × 10^−2^−1 × 10^−9^	1 × 10^−10^	∼5	This work
Tetrazolium–triiodomercurate	55.5 ± 0.4	1 × 10^−3^−6 × 10^−6^	4 × 10^−6^	30–50	[12]
N,N′-bis(Salicylaldehyde)-phenylenediamine	58.8 ± 0.3	3.2 × 10^−7^−3.2 × 10^−4^	1.5 × 10^−7^	≥10	[13]
Diamine donor ligand	25 ± 0.1	1.25 × 10^−5^−1.0 × 10^−1^	8.9 × 10^−6^	10	[16]
1-(2-Ethoxyphenyl)-3-(3-nitrophenyl)triazene	29.3 ± 0.2	1.0 × 10^−4^−5.0 × 10^−9^	2.5 × 10^−9^	∼5	[19]
Bis[5-((4-nitrophenyl)azo salicylaldehyde)]	30 ± 1	5 × 10^−2^−7 × 10^−7^	2.0 (±0.1) × 10^−7^	<10	[23]
4-(4-N,N-dimethylphenyl)-2, 6-diphenylpyrilium tetrafluoroborate	34	1.0 × 10^−8^−1.0 × 10^−3^	1.0 × 10^−8^	about 3 min	[28]
Ethyl-2-(benzoylamino)-3- (2-hydroxy-4-methoxyphenyl)-2-propenoate	48.5 ± 1.0	3.0 × 10^−7^−3.1 × 10^−2^	1.0 × 10^−7^	∼5	[37]
Substituted thiourea	28.4 ± 1.0	1.0 × 10^−7^−1.0 × 10^−1^	7.0 × 10^−8^	∼35	[38]
Cyclodextrins	20	0.9 × 10^−7^−1.0 × 10^−1^	0.9 × 10^−7^	20	[39]
N,N-dimethylformamide-salicylacylhydrazone	29.6	6.2 × 10^−7^−8.0 × 10^−2^	5.0 × 10^−7^	<30	[40]
2-[10-[(*E*)-2-(Aminocarbothioyl)hydrazono]-1,4-dihydroxy-9(10*H*)-anthracenyliden]-1hydrazinecarbothioamide	30.3	1.0 × 10^−7^−1.0 × 10^−2^	7.9 × 10^−8^	15	[41]
5,11,17,23-Tetra-*tert*-butyl-25,27-dihydroxy-26,28-bis(O-methylglycylcarbonylmethoxy) thiacalix[4]-arene	29.5	5.0 × 10^−8^−1.0 × 10^−2^	1.0 × 10^−8^	10	[42]

**Table 4.** Potentiometric determination of Hg (II) in water samples using proposed electrode and ICP.

Sample ^a^	Hg (II) (mg·L^−1^) ^b^			

	Proposed Electrode	ICP	RSD%	Recovery%
(1)	1.353	1.363	0.54	99.2
(2)	1.472	1.443	1.39	102.0
(3)	1.483	1.499	0.74	99.0
(4)	1.408	1.404	0.18	100.3

a From some ground water wells in the Gaza Strip;

b Mean data for three replicate measurements.
